# Contextualizing the Conscientiousness Index Tool and Correlating Burnout with Conscientiousness Index as a measure of Professionalism in Postgraduate Students

**DOI:** 10.12669/pjms.41.3.10906

**Published:** 2025-03

**Authors:** Shamila Tasneem, Usman Mahboob, Lubna Ansari Baig, Rehan Ahmad Khan

**Affiliations:** 1 Shamila Tasneem, MBBS, FCPS, CMT, MME. Senior Registrar, Department of Obstetrics & Gynecology, Allama Iqbal Medical College, Lahore. University of Lahore, Pakistan; 2 Usman Mahboob, MBBS, MPH, DHPE (UK), FHEA (UK). Fellow FAIMER (USA), Professor, Institute of Health Professions, Education & Research, Khyber Medical University, Peshawar, Pakistan; 3 Lubna Ansari Baig, MBBS, MPH, MMED, FCPS, PhD. Director PhD program, University College of Medicine & Dentistry, University of Lahore, Pakistan; 4 Rehan Ahmad Khan, MBBS, FCPS, FRCS, MME, MSc, PhD. Dean of Riphah Institute of Assessment and HOD Professor of Surgery Islamic, International Medical College, Riphah International University, Islamabad, Pakistan

**Keywords:** Burnout, Conscientiousness, Conscientiousness index, Postgraduate students, Postgraduate medical education, Professionalism

## Abstract

**Objective:**

To contextualize the Conscientiousness Index and determine the relationship between burnout and the Conscientiousness Index as a measure of professionalism in postgraduate students.

**Methods:**

This mixed-method study was done in two phases in Lahore from September 2023 to July 2024. The first phase involved contextualization of the Conscientiousness Index (CI) tool. A literature review, feedback from consultants and staff, identification of data sources, expert validation, and cognitive pretesting were conducted to contextualize the CI tool. In the second phase, a correlational study was done by collecting data using a purposive sampling technique (n=134). The CI scores were calculated from administrative records and clinical staff input. Burnout levels were assessed using the Maslach Burnout Inventory, which has three main constructs: Emotional exhaustion (EE), depersonalization (DP), and personal accomplishment (PA). Burnout and CI scores were compiled and analyzed using SPSS v26.0. The t-test, one-way ANOVA, Chi-square test, and Pearson’s correlation were used for data analysis.

**Results:**

A six-item CI scale was contextualized with S-CVI (0.89) and CVR >0.62. Mild burnout was found in 63 (47%) participants, while 71 (53%) exhibited moderate-to-severe burnout. The overall mean CI score was 42.1 ± 5.96 (total score: 50). No significant association was found between burnout and CI scores. However, a moderate negative correlation was observed between EE and CI scores (r=-0.69), and a weak positive correlation (0.19) was found between PA and CI scores, which was statistically significant (p = 0.02).

**Conclusion:**

The CI tool had good validity in the local context. There was a high prevalence of burnout among postgraduate students. No statistically significant association was found between burnout severity and CI scores. However, a weak correlation was found between burnout domains and CI scores. Further research is required to understand this weak correlation and validation of CI tool in different settings.

## INTRODUCTION

Burnout syndrome has a high prevalence in all healthcare professionals globally, but it is severe in postgraduate students.^1^ Burnout may vary in various specialties.^1^ Sulaiman et al. reported that 25% to 60% of the residents experienced burnout across worldwide studies.^1^ Burnout is not only associated with poor mental well-being but also with an increased incidence of anxiety, depression, and suicidal ideation among residents.^2^ Multiple contributing factors such as working hours, sleep deprivation, socioeconomic status, and various departments’ working environment make it challenging to address the residents’ wellness and burnout.^3^ Institutions use several interventions like wellbeing committees, stress and time management workshops, mindfulness training, and online physician wellbeing programs to improve residents’ mental well-being, but implementing these strategies is difficult.^4^ Despite the increasing incidence of burnout worldwide, it has been underrated in our settings.

Professionalism is a multi-dimensional construct and core competency of residency programs.^5^ Healthcare institutes use various tools such as Miller’s performance level, P-Max, LAMPS, and EPA to assess professionalism.^5^ Conscientiousness as a personality trait has many facets and relates to burnout and professionalism as well.^6^ As a high-order domain, it has six predominant facets: dutifulness, orderliness, cautiousness, striving for achievement, self-efficacy, and self-discipline.^6^ The Conscientiousness Index (CI) is a tool that uses objective data from residents’ activities at the workplace (for example: uninformed absence from work, lack of documentation, etc.) as a measure/proxy of their professionalism.^7^

It was developed by John C. McLachlan in 2009 for undergraduate medical students initially and later on used in postgraduate students of anesthesiology by collecting data from routine tasks expected to be performed by the residents at the workplace.^7^ Original CI tool components for anesthesia residents were sickness/absence, audit meeting attendance, appraisal documentation, and short notice requests.^8^ Digital media and literature have a high volume of material on the declining professionalism of resident doctors. Ensuring professionalism in postgraduate training requires constant monitoring and evaluation of residents’ professional conduct.^9^

One of the major challenges in this regard is the unavailability of a suitable tool to measure it.^9^ Majority of the tools used to assess professionalism are subjective.^10^ There is a continuous need to obtain more objective supporting data tools to assess professionalism. Limited studies have explored the association between burnout and professionalism in the local context, highlighting a significant gap. The objective was to bridge this gap by contextualizing the CI tool and determining the relationship between burnout and the conscientiousness index as a measure of professionalism in postgraduate students.

## METHOD

A Sequential exploratory mixed methods study was conducted from September 2023 to July 2024. In the first qualitative phase, contextualization of the CI tool was performed through expert validation, following the steps of the AMEE guide 87.^11^,^12^ A literature search was conducted for existing evidence of the CI tool, data collection points, and identify studies for adaptability in different contexts and prepare questions for experts. Seven experts were briefed about the study and CI tool. Discussion was generated based on open-ended questions for the identification of components of the tool according to local context and notes were taken. Experts were selected by purposive sampling having a postgraduate degree in any specialty with five years of teaching experience. Objective data sources were identified, and components of the CI were agreed upon for item modification. Expert validation was performed in two rounds.

### Ethical Approval:

It was obtained from the ethical committees of the three institutions: Institute-I (ERC 137/23/12-UOL); Dated: December 12, 2023, Institute-II (ERB158/9/11-01-24/S1-AIMC); Dated: January 11, 2024, and Institute-III (IRB/2023/1254/SIMS); Dated: January 23, 2024. I

In the first round, a nine item tool was sent to 15 experts via Google Forms for relevance and clarity, and the content validity index (I-CVI, S-CVI/Ave, S-CVI/UA) was calculated. In the second round, paper-based forms were distributed to the same experts. Ten experts responded and the content validity ratio (CVR) was determined by using the Lawshes formula.^13^ Cognitive pretesting was done with seven PGR’s administrative in charge to check the response process validity using verbal probing. Data were analyzed based on predefined criteria which included no change, rephrasing, difficulty to interpret, and omission from the scale. After contextualization of the CI tool, components were attendance, Sickness/absence, documentation, logbook entries, teaching presentations, and Inquiry/report about unprofessional behavior.

In the second quantitative phase, a correlational study was conducted using purposive sampling. A sample size of 134 was calculated by G*Power using two tails with a 5% margin of error, 95% confidence interval, and effect size of 0.3. After obtaining informed consent and ensuring anonymity, CI data were obtained from departmental administrative in-charges and clinical staff, based on data from the last six months. Trainees received a baseline score of 50 points, with points deducted for non-completion of tasks to avoid negative marking at the end.

Residents’ burnout levels were assessed using the Maslach Burnout Inventory (Christina Maslach & Susen), which includes three domains: emotional exhaustion (EE), depersonalization (DP), and personal achievement (PA).^14^ Responses were recorded on a Likert scale (0-6), with cut-off scores for postgraduate residents defined as EE: low<17, moderate 18-29, high>30; DP: low<5, moderate 6-11, high>12; and PA: low<33, moderate 34-39, high>40. High burnout was defined as high EE and DP and low PA.

The CI and burnout scores were manually calculated and compiled into an Excel spreadsheet with all identifying elements removed to maintain the anonymity of the residents. The data was exported to SPSS version 26.0 for statistical analyses. Demographic data are represented as frequencies and percentages for categorical variables and mean ±SD for continuous variables. T-tests and one-way analysis of variance (ANOVA) were used to compare demographic variables against burnout and CI scores, whereas the Chi-square test was used to determine the relationship between categorical variables. Pearson’s correlation calculated the correlation between burnout and CI scores, with p<0.05 considered significant.

## RESULTS

A nine-item scale was developed initially from the available literature and after the feedback from the experts. Experts agreed on a six-item scale with S-CVI (0.89), S-CVI/UA (0.75), and CVR >0.62. Items with I-CVI less than 0.7 and CVR <0.62 were removed from the scale. Seven PGR’s administrators checked the scale for the response process validity and no changes were suggested in verbal probing during cognitive interviews. Subsequently, a six-item tool was finalized. (Table-I).

**Table-I:** Components and scoring of the Conscientiousness Index (CI).

Component	Notes	CI points
Attendance	More than two leaves per month	-1 for each month
Sickness/Absence	Absent from the workplace without informing the department	-10 for each incident
Documentation	Daily notes on the requested timescale and complete	0 if complete and on time
		-5 if not complete or not on time
		-10 if not complete and not on time
Logbook entries	Regularly enter the logbook each month	-1 if not entered for each month
Presentations	Follow teaching roster	-1 if not appear for presentation
Inquiry/report about unprofessional behavior	Complaints from patients, colleagues, etc.	-1 for each occasion

Key descriptive statistics describing the resident population are shown in Table-II. In general, most of the residents were of similar age, as demonstrated by a comparatively narrow standard deviation of 1.83, with a mean age of 29.4 years.

**Table-II:** Demographic Data of the study population (n=134).

Characteristics	N (%)
*N*	134 (100%)
***Gender***	
Male	36 (26.9%)
Female	98 (73.1%)
***Marital Status***	
Married	86 (64.2%)
Unmarried	48 (35.8%)
***Year of residence***	
1^st^ Year	30 (22.4%)
2^nd^ Year	50 (37.3%)
3^rd^ Year	20 (14.9%)
4^th^ Year	28 (20.9%)
Final Year	6 (4.5%)

Prevalence of mild and moderate to severe burnout was found in 47% and 53% of the residents, respectively. No statistically significant association was found based on age, gender, marital status, and year of residency. The results of the bivariate analysis are summarized in Table-III. There was no significant association between the degree of burnout and CI score. The overall mean CI score was 42.1±5.96, with minimum and maximum values of 25.0 and 50.0, respectively. The highest mean CI score was calculated for radiology residents at 47.7 ±3.03, and the lowest for psychiatry residents at 35.9±5.5. There was a statistically significant association (p < 0.001) between the CI score and training specialty. No significant association was found based on gender, marital status, year of training, and institute.

**Table-III:** Comparison of burnout with CI score.

CI score	Burnout		

	Mild	Moderate	Severe
N	63	34	37
Mean	42.6	41.7	41.1
SD	7.71	5.15	6.28
F value (P value)	0.56(0.567)		

The correlation between burnout domains and CI scores is summarized in Table-IV. There was a moderate negative (-0.69) but statistically insignificant association between emotional exhaustion and CI score and a weak positive (0.19) but statistically significant association (p=0.02) between personal accomplishment and CI score.

**Table-IV:** Pearson correlation coefficients between burnout and CI score.

Burnout Dimension	CI score	P value	Significance
Emotional Exhaustion	-0.69	0.42	Insignificant
Depersonalization	-0.084	0.33	Insignificant
Personal Accomplishment	0.19	0.02	Significant*

## DISCUSSION

The present research aimed to investigate the association between burnout and the Conscientiousness Index as a measure of professionalism. It highlights the prevalence of burnout in PGRs. Contrary to the expectation, no significant correlation was found between burnout and the Conscientiousness Index. No significant association was found based on gender, marital status, and year of residency, but statistically significant high burnout was reported in surgical and allied specialties. The findings demonstrate that burnout is a significant issue among postgraduate residents, with 71(53%) of them experiencing moderate-to-severe burnout. Slayer et al. in a meta-analysis found that burnout in healthcare professionals is associated with a range of negative outcomes, including increased medical errors, poor patient satisfaction, and reduced empathy, all of which can adversely impact patient care.^15^

These findings are in line with the study conducted by Thrush et al. in USA reported moderate to high levels of personal burnout (49-52%) in PGRs with more prevalence in females.^16^ Another study conducted in Pakistan by Kamran et al. observed that dental postgraduate residents and dental faculty dealt with moderate burnout, but residents were more affected as compared to dental faculty.^17^ Our study found a high prevalence of burnout in residents of public sector hospitals as compared to private sector hospitals. A study conducted in Jordan by Nimer et al. in 2021 also found high levels of burnout in public-sector hospitals compared to the private sector.^18^ These findings may be due to high workload, poor working environment, sleep deprivation, and logistical challenges at public sector hospitals.

We contextualized the CI tool and used it effectively for PGRs. Our study found that the correlation coefficient between burnout domains and CI score showed a moderate negative correlation between EE and DP which is statistically insignificant, while the PA domain has a weak positive but statistically significant correlation with CI score (p=0.02). However, an earlier study found a significant correlation between burnout and lower chances of meeting professional expectations in residents.^19^ To improve the EE score, strategies such as a conducive work environment, open communication, mentoring, and flexible duty hours may be used.^20^,^21^

We found high CI scores across different specialties and years of residency. This might be because we deal with a highly conscientious group of future consultants. The CI scores showed significant differences among specialties with the highest CI score in residents of Radiology, followed by Surgery, Obstetrics and Gynecology, Medicine, and the lowest in Psychiatry residents but no significant differences among years of residency, gender, or marital status. Limited CI data are available for comparison in resident settings. Other factors may affect this relationship, including socioeconomic status, number of children, housing issues, and work environment. An in-depth exploration of these factors may provide insights.^22^,^23^ As no correlation was found between CI score and burnout it might be due to the “failure to fail” phenomenon in the collection of CI data.^8^ Reasons for this phenomenon may include a lack of proper documentation, confusion about what to document, difficulty compiling data from different sources, and the potential consequences of using this data.^8^ Professionalism among postgraduate residents is a massive stake due to its critical debt and may need to include more sub-facets of conscientiousness.

### Strengths & Limitations:

We used validated tools and contextualized CI tool which increased its relevance and impact. This study also has a few limitations. The data was collected from multiple sources, leading to logistical challenges, as it was not compiled at a single point. Additionally, data collection was limited to three institutions, which restricts the generalizability of the results to similar contexts only.

### Future Directions:

It is recommended to add additional data points to the CI tool in which trainees demonstrate higher levels of conscientiousness to better explore the association between these two variables. This study serves as a foundation for future longitudinal research with a larger sample size. The CI tool can be further validated by applying it across different settings to assess its effectiveness in measuring professionalism.

## CONCLUSION

This study contextualized and utilized the Conscientiousness Index (CI) tool to examine the relationship between burnout and professionalism in PGRs. Although high burnout levels were prevalent, no significant association was found between burnout severity and CI scores. A positive correlation with personal accomplishment and a negative correlation with emotional exhaustion and depersonalization with CI scores were observed. Further large-scale studies need to be conducted to investigate the causes of this weak association and validation of CI tool across different settings.

### Author’s Contribution:

**ST:** Conceived, designed, data collection, did statistical analysis, writing, and edited the manuscript.

**UM:** Supervised the thesis and was involved in it from its inception.

**LAB:** Co-supervised the thesis and gave final approval.

**RAK:** Reviewed and approved the manuscript.

All authors have approved the final version and are accountable for integrity of the study.
